# CARD-FISH for Environmental Microorganisms: Technical Advancement and Future Applications

**DOI:** 10.1264/jsme2.ME12107

**Published:** 2012-10-31

**Authors:** Kengo Kubota

**Affiliations:** 1 Department of Civil and Environmental Engineering, Tohoku University, 6–6–06 Aoba, Aramaki, Aoba-ku, Sendai, Miyagi 980–8579 Japan

**Keywords:** catalyzed reporter deposition (CARD), fluorescence *in situ* hybridization (FISH), single cell detection, tyramide signal amplification (TSA)

## Abstract

Fluorescence *in situ* hybridization (FISH) has become a standard technique in environmental microbiology. More than 20 years have passed since this technique was first described, and it is currently used for the detection of ribosomal RNA, messenger RNA, and functional genes encoded on chromosomes. This review focuses on the advancement and applications of FISH combined with catalyzed reporter deposition (CARD, also known as tyramide signal amplification or TSA), in the detection of environmental microorganisms. Significant methodological improvements have been made in CARD-FISH technology, including its combination with other techniques and instruments.

## Introduction

Since it was first developed (20), fluorescence *in situ* hybridization (FISH) has become one of the most routinely used molecular techniques in environmental microbiology. FISH can be used to detect, identify, and enumerate environmental microorganisms without requiring culture, and therefore it has been used to help elucidate the microbial ecology of many habitats, including soil, sediments, aquatic environments, and engineered sludge (reviewed in refs. 7, 8, 53). Nevertheless, there are several problems in the application of FISH, primarily insufficient sensitivity due to the low number of target molecules in cells, low probe permeability of cells, and poor probe hybridization efficiency (7). Many methods have been devised to overcome these problems (reviewed in refs. 9, 86, 88). This review will focus on the technical advancement and applications of a sensitive FISH technique, catalyzed reporter deposition (CARD)-FISH, also known as tyramide signal amplification (TSA)-FISH (Table 1). The applications of CARD-FISH will be discussed, not only in rRNA-targeted phylogenetic identification but also in linking microbial phylogeny to physiology and metabolic activity.

## Catalyzed Reporter Deposition: CARD

**Fig. 1:**
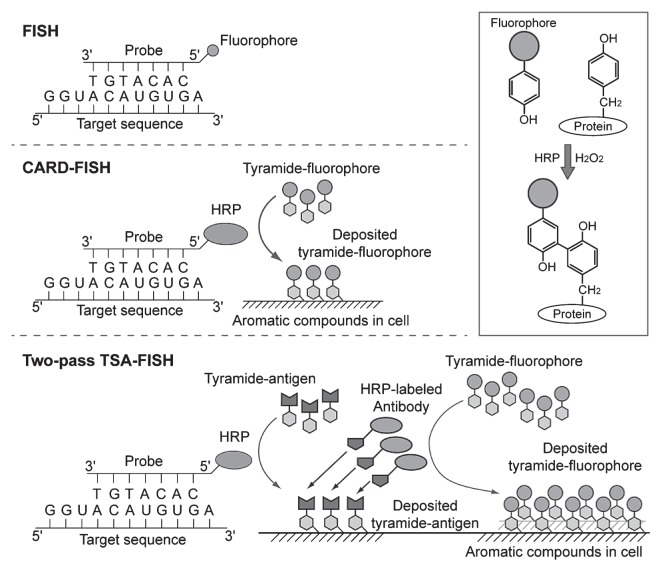
Schematic depiction of FISH, CARD-FISH, and two-pass TSA-FISH. The scheme in the box depicts tyramide immobilization on tyrosine.

CARD was first reported more than twenty years ago as a novel method of signal amplification for immunoassays and membrane immunoassays (15, 16), and was later applied to FISH (33, 66). The principle of CARD is as follows: in the presence of hydrogen peroxide, horseradish peroxidase (HRP) converts tyramide into a radical intermediate. This radical tyramide nonspecifically reacts with aromatic compounds, such as tyrosine and tryptophan, in cells or blocking reagents (Fig. 1). This radical reaction occurs only near the HRP molecule and on a very short timescale. As a result, a great number of tyramides are deposited around the HRP molecule. Tyramides with conjugates (*e.g.*, fluorescein, cyanine dyes, Alexa fluor dyes, biotin, etc.) for the CARD amplification system are commercially available from several companies. Alternatively, tyramide conjugates can be prepared by simple and rapid chemical reactions (27).

## Fundamental developments in CARD-FISH

The first application of CARD-FISH in environmental microbiology was reported by two different groups in 1997. Schönhuber *et al.* (68) reported a direct method using HRP-labeled probes (Fig. 1), while Lebaron *et al.* (39) described an indirect method using biotinylated probes and HRP-labeled streptavidin. Both studies showed significant signal amplification after the CARD reaction, with more than 10-fold stronger signals than mono-fluorescently labeled probes. The direct method is simpler than the indirect method as it omits the immunological reaction step and is therefore more popular in environmental microbiology.

### Improving sensitivity and reducing background

Many strategies have been adopted to further improve CARD-FISH signals, mainly by amending the CARD working solution. The addition of 10–30% dextran sulfate has positive effects on signal localization (82) and signal intensity (37). This is attributed to the effect of volume exclusion, a result of the trapping of solvent water molecules by long polymer rods (83); however, dextran sulfate sometimes introduces spotty background signals dispersed over the entire slide (82). This problem is overcome by washing at elevated temperatures (45–60°C) (30, 82). The addition of an inorganic salt and/or an organic reagent enhances CARD-FISH signals (17). Inorganic salts include NaCl, MgCl_2_, KCl, CaCl_2_, sodium phosphate, sodium acetate, ammonium acetate, and ammonium sulfate. Most preferably, the concentration of the inorganic reagent ranges from at least 2 M to saturation. Preferred organic reagents are described in the paper (17): the preferred enhancer for non-fluorescent reagents is N-(5-hydroxypentyl)-3-(*p*-hydroxyphenyl) propionamide and that for fluorescent reagents is *p*-iodophenyl boronic acid. The concentration of the organic reagent ranges from approximately 1×10^−6^ to 1×10^−4^ M. This discovery was unexpected because organic reagents act as inhibitors rather than as enhancers (17), and the detailed mechanisms are not fully understood. For application to environmental micro-organisms, the addition of NaCl (2 M) and *p*-iodophenyl boronic acid (at 20 times the tyramide concentration) to the CARD working solution with fluorescently labeled tyramides has been reported (29, 59). The concentration of tyramide in the working solution is also an important factor. Detection rates for environmental bacteria were increased by elevating tyramide concentration in the working solution; however, too much tyramide causes high background fluorescence (56).

To minimize background fluorescence, the concentration of HRP-labeled probes is an important factor. Many FISH protocols use fluorescently labeled probes at concentrations of 0.5–1 μM or 2–5 ng μL^−1^ (3, 6, 36, 46, 61); however, these probe concentrations resulted in nonspecific fluorescent signals following CARD-FISH (56). To reduce the background, lower probe concentrations (0.1 μM or 0.5 ng μL^−1^) were used for CARD-FISH. Furthermore, a blocking reagent is often involved in the CARD working solution to minimize nonspecific fluorescent deposition (72).

Two-pass TSA-FISH is another approach for enhancing signal intensity (Fig. 1). Two-pass TSA-FISH involves two sequential TSA reactions. In the first reaction, dinitrophenyl (DNP)-labeled tyramide is deposited instead of a fluorophore-labeled tyramide. After incubation with an HRP-conjugated anti-DNP-antibody, the second TSA reaction is carried out with a fluorophore-labeled tyramide. This technique was first applied to eukaryotic cells (81) and later to prokaryotic cells (37). In two-pass TSA-FISH, the addition of both dextran sulfate and blocking reagent is especially important to maximize signal intensity and minimize nonspecific staining (37).

### Permeabilization

Because HRP is approximately 5–6 nm with a molecular weight of approximately 40 kDa, probes labeled with this large molecule penetrate fixed cells very poorly compared with fluorescently labeled probes (molecular weights of fluorochromes are generally 500–1,000 Da); therefore, before applying CARD-FISH, permeabilization protocols must be optimized for the group of interest. This is often laborious because the range for “optimum” permeabilization is very narrow. Even though some prokaryotic species can be detected without any treatments, *e.g.*, *Planctomycetes* in marine sediments (29) and methanogens with an s-layer (38), most prokaryotic cells need to be pretreated for probe penetration.

Optimization of the fixation procedure is the first step in optimizing the permeabilization process. Fixation with protein denaturing reagents (*i.e.*, ethanol fixation) typically produces better permeability than that with cross-linking reagents (*i.e.*, paraformaldehyde fixation) (68); however, some prokaryotes exhibit a loss of fluorescent signals following ethanol fixation (38). The concentration, fixation time, and temperature are also important. Pizzetti *et al.* (64) reported that a higher detection rate for *Planctomycetes* was obtained by FISH than by CARD-FISH when samples were fixed with 2% formaldehyde, but the opposite results were obtained when samples were fixed with 1% paraformaldehyde. Furthermore, storage conditions and term also affect the permeability. Long-term storage of samples resulted in higher detection rates because permeability inexplicably increased during storage (38, 93).

Prior to permeabilization, cells are immobilized on slides or filters using low-melting point agarose to prevent major cell loss during permeabilization and CARD-FISH (9, 56). In the first report on agarose embedding, no bacterial cell loss was observed even after stringent lysozyme treatment (10 mg mL^−1^ for 90 min at 37°C) when samples were embedded in agarose (56). Agarose embedding is currently included in the majority of CARD-FISH protocols.

Enzymatic treatments using lysozyme, achromopeptidase, proteinase K, and pseudomurein endopeptidase are often employed for permeabilization. Lysozyme is the most commonly used enzyme for treatment as it catalyzes the hydrolysis of 1,4-beta-linkages between *N*-acetylmuramic acid and *N*-acetyl-D-glucosamine residues in peptidoglycans. Many Gram-negative bacteria are permeabilized by lysozyme treatment (*e.g.*, 42, 55, 56); however, because several Gram-positive cultures are resistant to lysozyme (18), and because lysozyme only partly digests the murein multilayers of fixed Gram-positive cells (72), some Gram-positive bacteria cannot be permeabilized by lysozyme treatment alone. Simultaneous treatment with achromopeptidase and lysozyme is effective for lysis of these Gram-positive bacteria (11, 22). Sekar *et al.* (72) introduced achromopeptidase treatment following lysozyme treatment for permeabilization of Gram-positive *Actinobacteria*. Interestingly, this achromopeptidase treatment is only effective when conducted after lysozyme treatment, but not before (72). Achromopeptidase hydrolyzes lysyl peptide bonds (79), and lysozyme treatment likely improves the accessibility of achromopeptidase to the peptide bonds. This treatment is used for permeabilization of many microbial cells (*e.g.*, *Planctomycetes* [64, 90] and *Archaea* [29]). Proteinase K is also used in many protocols. Although some studies have found that proteinase K treatment is difficult to control and causes unstable results (55, 69), this treatment is effective for many *Archaea* (38, 42, 47, 65, 75). Pseudomurein endopeptidase is effective for permeabilization of methanogens with pseudomurein. The glycan strands of pseudomurein consist of alternating β(1→3)-linked N-acetyl-D-glucosamine and N-acetyl-L-talosaminuronic acid residues, instead of the N-acetyl-D-muranosamine of murein and thus, the pseudomurein cell wall structure is resistant to the three enzymes described above, as well as to many other enzymes (84). Pseudomurein endopeptidase was originally isolated from (pro)phages in *Methanothermobacter wolfei* (PeiW) (44) and *Methanothermobacter marburgensis* (PeiP) (63), and the enzyme-encoding genes have since been cloned (45). Treatment with recombinant PeiW effectively permeabilized fixed methanogens with pseudomurein for FISH (52) and CARD-FISH (31, 38) applications.

Chemical treatments are also used for permeabilization. Sodium dodecyl sulfate (SDS) is a detergent usually used in hybridization buffer to improve probe accessibility to rRNA (12). In addition to this purpose, increased concentration of SDS (*e.g.*, 1%) in hybridization buffer successfully permeabilized *Methanotorris igneus* (formaly *Methanococcus igneus*) for penetration of HRP-labeled probes (5). Furthermore, HCl treatment is effective for marine *Archaea* (47, 90). SDS or SDS/dithiothreitol treatment followed by lysozyme treatment has also been reported (19, 54).

Microwave treatment has been applied for the detection of *pmoA* (the alpha subunit of the particulate methane monooxygenase) mRNA in tissue sections of *Bathymodiolus puteoserpentis* (59), and for the detection of ANME-2c-group organisms in marine sediment (60). This type of physical treatment is preferable to enzymatic treatments, especially when the cell structures and effective permeabilizers are unknown. Very recently, microwave treatment in Tris-EDTA buffer (pH 9.0) was reported for the detection of *Bacteria* and *Archaea* (77). It is suggested that Tris binds to lipopolysaccharides and replaces stabilizing Ca^2+^ and Mg^2+^ (80). EDTA removes these divalent cations by chelation and therefore the interaction between lipopolysaccharide molecules is reduced; however, Tris-EDTA permeabilization cannot stand alone. The authors noted that hybridization should also be conducted in a microwave oven, rather than a normal oven, for successful application (77).

### Endogenous peroxidase

The CARD reaction is initiated by the oxidation of hydrogen peroxide to the hydroxyl radical by peroxidase activity; therefore, endogenous peroxidase should be inactivated prior to hybridization. False-positive signals caused by endogenous peroxidase cannot be explained by the mere presence of peroxidase (55). Unfortunately, there is no comprehensive method to inactivate endogenous peroxidase activity in various environments and thus it should be optimized for each sample. Treatment with H_2_O_2_ in water or methanol is often used. Ishii *et al.* (29) reported that 0.15% H_2_O_2_ in methanol successfully inactivated endogenous peroxidase activities without decreasing the number of 4′,6-diamidino-2-phenylindole (DAPI)-stained cells, whereas higher concentrations (up to 3%) of H_2_O_2_ resulted in a significant decrease in the number of DAPI-stained cells (29). Our research group also found that CARD-FISH signals were significantly weaker following pre-treatment with higher concentrations of H_2_O_2_ (unpublished results). Nucleic acids are likely hydrolyzed by the strong oxidant H_2_O_2_. In contrast, the strong endogenous peroxidase activity in anaerobic ammonium oxidation (anammox bacterial cells needed to be treated with 3% H_2_O_2_ for sea samples (90) or 30% H_2_O_2_ for bioreactor samples (55). In addition, diethylpyrocarbonate (56) and HCl (72) treatments are also useful for inactivation.

### Thermal stability of HRP

It was traditionally thought that HRP should be handled below 42°C. Thus, hybridization of HRP-labeled probes is carried out at lower temperatures (*e.g.*, at 35°C or 40°C), rather than the standard temperature of 46°C, to prevent inactivation of HRP (37, 61). However, a more recent study investigated the thermal stability of the HRP molecule attached to oligonucleotides and found that HRP is not inactivated, even after hybridization at 55°C for 2 h and washing at 57°C for 30 min (29); therefore, conventional hybridization and washing temperatures (46°C and 48°C) can also be used. Nevertheless, hybridization stringency, *i.e.*, the formamide concentration, should be re-optimized for HRP-labeled probes because the dissociation profile of HRP-labeled probes differs significantly from that of fluorescently labeled probes (28).

## Phylogenetic staining

**Fig. 2:**
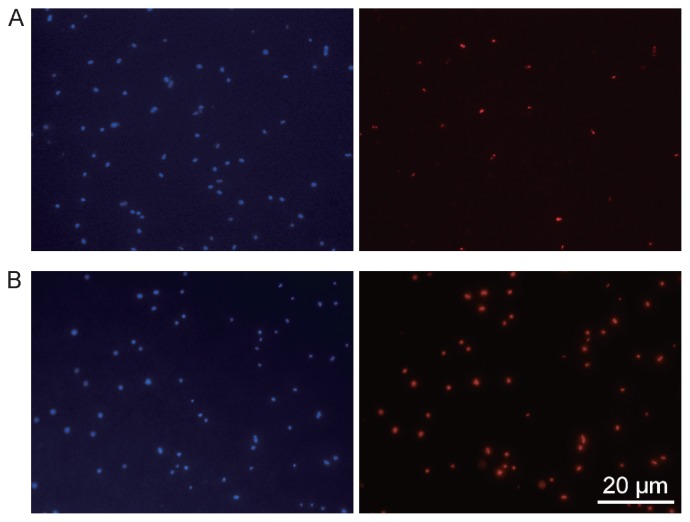
Detection of marine bacteria by FISH with Cy3-labeled probes (A) and by CARD-FISH with the deposition of tyramide-Cy3 (B). Lysozyme treatment was carried out prior to hybridization. Exposure times were 1 s for FISH and 0.1 s for CARD-FISH. Stronger signals and higher detection rates were obtained after CARD-FISH. Left (DAPI-stained cells) and right (FISH signals) epifluorescent micrographs show identical fields.

CARD-FISH has been used for phylogenetic staining of microorganisms in many environments, including marine, freshwater, soil, sediment, and engineered sludge (*e.g.*, 19, 23, 25, 26, 29, 34, 38, 42, 47, 54–56, 64, 65, 67, 72, 75, 90, 91), and a basic protocol for CARD-FISH target to rRNA is described in Table 2. Microorganisms living in oligotrophic environments often have a low rRNA content; hence, CARD-FISH is especially superior to FISH under these circumstances because of its high sensitivity. For example, significantly higher detection rates of marine bacteria were achieved by CARD-FISH (85–100%) compared to FISH using Cy3-labeled probes (19–66%) (56) (also see differences in Figure 2). Similar results were seen for bacteria in sediment (29, 56) and freshwater (72). Conversely, CARD-FISH detection of bacteria in activated sludge (68), anammox cells in marine environments (90), methanogenic archaea in anaerobic granular sludge (38), and *Planctomycetes* in marine water (64) showed lower detection rates than those obtained by FISH. As described above, permeabilization is always an issue for application of CARD-FISH, and detection rates using CARD-FISH may be lower than those using FISH when the target organisms contain sufficient rRNA for conventional FISH detection.

Strong CARD-FISH signals also enable reliable detection of microorganisms with autofluorescence. Cyanobacteria have yellowish-red autofluorescence and are difficult to detect using FISH with fluorescently-labeled probes (69). CARD-FISH with fluorescein-labeled tyramide successfully overcame the background (autofluorescence) noise of cyanobacteria (1, 69, 89).

Phylogenetic staining that targeted tmRNA (transfer-messenger RNA) was also developed to overcome the limitations of phylogenetic classification using the rRNA gene (*i.e.*, poor resolution of closely related strains, subspecies) (70). The tmRNA molecule was first discovered in *Escherichia coli* (40). It is a stable RNA that is present at fewer than 1,000 copies per cell. Because of its low copy number, tmRNA is difficult to detect using mono-fluorescently labeled probes, but it can be detected successfully with CARD-FISH and can be used as a marker for phylogenetic analysis (70).

## Functional identification

The number of target molecules required to detect microorganisms by CARD-FISH with HRP-labeled oligonucleotide probes has been quantified, and the detection limit was found to be dozens of molecules (28); therefore, highly-expressed mRNA can be detected by CARD-FISH, but genes or mRNA with low expression cannot. Further signal amplification should be performed for the detection of mRNA and genes by CARD-FISH. Two-pass TSA-FISH, as described above, is one way to increase signal intensity.

Alternatively, the use of multiply-labeled polynucleotide probes instead of oligonucleotide probes is an option, but their characteristics are different (Table 3). Polynucleotide probes are typically generated by PCR (double-stranded DNA [dsDNA] polynucleotides) or reverse transcription (RNA polynucleotides). dsDNA and RNA polynucleotide probes are often used to detect genes and mRNA, respectively. Dozens of haptens or fluorophores are incorporated into the polynucleotides during synthesis, resulting in higher sensitivity than single-molecule-labeled oligonucleotides. Nevertheless, the specificity of polynucleotide probes is lower than that of oligonucleotide probes. Oligonucleotide probes can distinguish single base mismatches, although it depends on the sequence and the position of the mismatch (3). Even if single base mismatches are not distinguishable, the use of competitor probes makes this possible (29, 37, 42, 46). Polynucleotide probes can also distinguish closely related species under very stringent hybridization conditions; however, the signal intensity is significantly decreased under these conditions, diminishing the merits of the polynucleotide probe (78). This difference in specificity is a result of the difference in hybridization behavior between oligonucleotides and polynucleotides. For example, the melting temperature of an oligonucleotide is sequence-dependent, whereas that of a polynucleotide is base content-dependent (76). The threshold for discrimination using polynucleotide probes has been reported to be in the range of 70–89% of sequence identity (24, 31, 43); therefore, polynucleotide probes are frequently used for phylogenetic discrimination at higher taxonomic levels (21). The sequence identities of functional genes within a genus are often low. In the case of the *mcrA* (the alpha subunit of the methyl-coenzyme M reductase) gene, the average nucleotide sequence identities within a genus and family are only 88.9% and 79%, respectively (74); therefore, polynucleotide probes are useful for functional staining. Recently, Moraru *et al.* (49) proposed a concept and software for polynucleotide probe design. Another drawback of polynucleotides is lower permeability than oligonucleotides because of differences in size. In addition, design flexibility is lower, and polynucleotide probes are not commercially available.

### mRNA-FISH

The detection of mRNA is particularly challenging because these molecules are unstable and only present at very low copy numbers. Preparation of control samples is the first highest hurdle. Clone-FISH, which was originally developed for rRNA-FISH (71), is often used for the optimization of hybridization conditions for both oligonucleotide probes (37) and polyribonucleotide probes (58) for mRNA-FISH. The first report of the detection of mRNA by CARD-FISH was in 1998 (85). In that study, digoxigenin (DIG)-labeled RNA polynucleotide probes and HRP-conjugated anti-DIG-antibodies were used to detect *iap* (gene encoding invasion-associated protein) mRNA in *Listeria monocytogenes* cells. Four years later, *nahAc* (naphthalene dioxygenase) mRNAs in bacteria in contaminated groundwater were detected using biotinylated oligonucleotide probes and HRP-labeled streptavidin (10). These authors introduced DNase treatment prior to hybridization to improve the accessibility to mRNA, and extended exposure times (up to 10 s) to acquire digital images of fluorescent signals. The weak signals were overcome by the development of two-pass TSA-FISH (37). Archaeal *mcrA* mRNA was detected with intensified signals using HRP-labeled oligonucleotide probes, and the discrimination of single-base mismatches was also achieved using competitor probes. Recently, mRNA-FISH combined with microsensor measurements has been reported (35). This method has the potential to connect microenvironments and microbial activities as defined by mRNA expression.

Linking physiology and phylogeny using simultaneous mRNA and rRNA staining is also an intriguing challenge. In 2004, the simultaneous detection of mRNA and rRNA was reported (58). In the protocol of Pernthaler *et al.* (58), mRNA-FISH targeting *pmoA* was performed using DIG-labeled RNA polynucleotide probes. After inactivating the HRP used for the first CARD reaction, rRNA-FISH was subsequently carried out using HRP-labeled oligonucleotide probes. It is better to perform mRNA-FISH first for the simultaneous detection of mRNA and rRNA because mRNA is typically present at a low copy number and is very unstable. Using this method, archaeal ammonia oxidizers in soil were quantified by simultaneous detection of archaeal *amoA* (the alpha subunit of the ammonia monooxygenase) mRNA and rRNA, and their high relative abundance among the overall archaeal community were demonstrated (65).

More recently, sequential mRNA FISH followed by fluorescence-assisted cell sorting (SmRFF) has been developed, and active nitrite reducers in an activated sludge sample were successfully identified (50). This technique is useful to identify phylogenetically unknown mRNA-expressers in environments.

### Gene-FISH

The detection of genes encoded on chromosomes has been developed recently (30, 31, 48). DNA is much more stable than mRNA, but the copy number of a given gene is usually very low. The detection of genes can provide insight into microbial functional potential, whereas the detection of mRNA demonstrates the transcriptional activities of functional genes in microbial cells. Gene detection using the TSA reaction was first reported by Kawakami *et al.* (30), who used two-pass TSA-FISH with oligonucleotide probes targeting the *mcrA* gene. Locked nucleic acid-incorporated DNA probes were used to improve hybridization efficiency and specificity (36, 92). These authors demonstrated that two-pass TSA-FISH can detect a single-copy gene on a chromosome with specificity sufficient to discriminate a single-base mismatch. The detection efficiency was approximately 15% when the single-copy gene was targeted; however, efficiency increased to more than 50% when a multi-copy gene, the rRNA gene, was targeted. Recently, a high detection efficiency method (more than 98%) has been developed, two-pass TSA-FISH with dsDNA polynucleotide probes, and applied to detect the *apsA* (the alpha subunit of adenosine-5′-phosphosulfate kinase) and *mcrA* genes in sludge samples (31).

The simultaneous detection of genes and rRNA was reported by Moraru *et al.* (48). The authors used CARD-FISH with dsDNA polynucleotide probes to detect the crenarchaeotal *amoA* gene in a seawater sample. The detection efficiency of this method was approximately 40%. To maximize gene detection efficiency, four dsDNA poly-nucleotide probes, targeting different sites on the target gene, were prepared and applied simultaneously (62).

In combination with other techniques, Kenzaka *et al.* (32) deposited biotinylated tyramide and subsequently applied nanogold-conjugated streptavidin. For further signal amplification, they employed gold enhancement and successfully detected the green fluorescent gene and ampicillin resistance gene on plasmids using a scanning electron microscope.

## Cell isolation

Fluorescence-activated cell sorting (FACS) is a popular method for cell isolation. CARD-FISH was first used for the isolation of marine picoeukaryotes (14). For these experiments, all procedures were performed using cells in suspension. Cells were concentrated by centrifugation at various steps of the experiment, *e.g.*, during washing. Using this method, cell loss can be avoided; however, the wide range of cell sizes makes it impossible to recover cells quantitatively by centrifugation. The *g* forces required to sediment small cells might damage larger cells (73), and cell concentration efficiency by centrifugation changes based on the properties of the buffer used (*e.g.*, salt concentration and viscosity), temperature, and other variables. As an alternative, a protocol for cell recovery following CARD-FISH using a membrane filter was developed (73). In this method, the agarose-embedding procedure is omitted because it interferes with the detachment of cells from filters, and agarose particles interfere with flow cytometric analysis. For cell detachment, filters were incubated in various solutions, followed by vortexing or sonication. The authors found that incubation in NaCl-Tween solution followed by vortexing was the best strategy for detaching cells from their seawater samples (cell recovery efficiency was approximately 70%). The ideal conditions may differ based on the sample of interest. Recently, cell sorting following mRNA-FISH was reported, as described above (50). In that report, mRNA-FISH was carried out for cells embedded in agarose on a glass slide, and the cells were recovered from the slide into suspension in pre-warmed PBS buffer.

An interesting method using CARD-FISH and paramagnetic beads for cell isolation (magneto-FISH) was developed recently to investigate the syntrophic association of cell aggregates (60). In magneto-FISH, fluorescein-labeled tyramide is deposited after the *in situ* hybridization of HRP-labeled probes. Subsequently, monoclonal mouse anti-fluorescein antibody-labeled pan-mouse paramagnetic beads are used to label the target cells with magnetic beads. The target cells, and cells associated with the target cells, can be separated from other cells using a magnet. Isolated cells can then be used for subsequent analyses (*e.g.*, sequencing and isotopic studies). Because tyramide is deposited on intercellular proteins as well as on cell surface proteins, the paramagnetic beads (5 μm in the aforementioned study) do not need to penetrate the small microbial cells. Magneto-FISH does not require any special instruments, is inexpensive, and can be used as a substitute for FACS.

## Linking metabolic activity and phylogeny

CARD-FISH can also be combined with techniques such as microautoradiography (MAR) and with instruments such as nanoSIMS (secondary ion mass spectrometry), which are used to evaluate microbial metabolic activities using isotopic or radioisotopic substrates. Unlike FISH-MAR, CARD-FISH-MAR can be applied to oligotrophic samples because of the greater sensitivity of CARD-FISH (26, 34, 75). A detailed technical description of this protocol is reviewed elsewhere (2).

More recently, CARD-FISH has been used for nanoSIMS analyses. The principles of nanoSIMS and its applications for environmental microbiology are reviewed elsewhere (87). Because nanoSIMS is not a microscope-based method, it cannot be used to observe fluorescent signals. In simultaneous isotopic measurements and phylogenetic identification of microbial cells by nanoSIMS, halogen elements such as iodine, fluorine, and bromine are often used. SIMSISH (SIMS *in situ* hybridization) uses 5′-iodo-2′-deoxycytidine instead of 2′-deoxycytidine in oligonucleotide probes (41); however, this method has limited sensitivity because the number of substitutable bases depends on the probe sequence. Enhanced elemental labeling (EL)-FISH (13) and halogen *in situ* hybridization (HISH) (51) were developed as more sensitive methods. Both of these techniques are based on CARD-FISH, but fluorochromes that contain a halogen element (*e.g.*, 544Br, BODIPY TMR-X, and Oregon Green 488-X) are coupled with tyramine and are used for the CARD reaction. Following CARD-FISH, a large number of halogen elements are deposited in cells, resulting in sensitive detection by nanoSIMS.

CARD has also been used to identify DNA-synthesizing bacteria in marine environments (57). Bromodeoxyuridine (BrdU) is a halogenated nucleotide analogue of tritiated thymidine and is incorporated during DNA synthesis in bacteria. After incubating cells in BrdU-containing medium, BrdU-incorporated cells were detected using HRP-labeled anti-BrdU-antibody and the CARD reaction.

## Conclusion

CARD-FISH is one of the most important molecular tools for enhancing our understanding of environmental micro-organisms. Because of its increased sensitivity and the many recent technical developments, CARD-FISH can now be used for the detection of not only rRNA, but also mRNA and genes encoded on chromosomes in microorganisms in various environments. This review describes the fundamental principle, applications, and problems of CARD-FISH; however, the application of CARD-FISH requires some technical advances to overcome several drawbacks, as described in this review (*e.g.*, low probe permeability and endogenous peroxidase activity). Further technical developments in CARD-FISH, including the combination with recent leading-edge technologies, will provide new insights into environmental microbiology with single-cell resolution.

## Figures and Tables

**Table 1:** Important technical developments in the history of CARD-FISH for environmental microorganisms

Year	Description	Reference
1989	• First CARD publication (for immunoassay)	15
	• Development of FISH for microorganisms	20
1991	• FISH applied to uncultured microorganisms	4
1997	• CARD-FISH for environmental microorganisms	39, 68
1998	• CARD-FISH with polyribonucleotide probes for mRNA detection	85
2002	• Low-melting point agarose-embedding protocol	56
	• Detection of DNA synthesizing (BrdU incorporated) bacteria by CARD-FISH	57
	• CARD-FISH with oligonucleotide probes for mRNA detection	10
	• Development of clone-FISH	71
2003	• CARD-FISH combined with FACS	14
2004	• Simultaneous detection of rRNA and mRNA by CARD-FISH with polyribonucleotide probes	58
	• CARD-FISH combined with microautoradiography	75
2006	• Two-pass TSA-FISH with oligonucleotide probes for mRNA detection	37
2008	• EL-FISH and HISH for nanoSIMS analysis	13, 51
	• Magneto-FISH for cell capturing	60
2010	• Two-pass TSA-FISH with oligonucleotide probes for gene detection	30
	• Simultaneous detection of rRNA and gene by CARD-FISH with polydeoxyribonucleotide probes	48
2012	• Two-pass TSA-FISH with polydeoxyribonucleotide probes for gene detection	31
	• mRNA-FISH combined with FACS	50

**Table 2:** Basic protocol for CARD-FISH for rRNA

***Cell fixation*** 1 Fix samples with paraformaldehyde or ethanol, and store in ethanol/PBS soltion at −20°C. ***Embedding*** *on glass slide* 2-1 Mix the samples with PBS, SDS (final conc., 0.001%), and low-melting point agarose (final conc., 0.1%), apply appropriate amount to each well, and dry at 35–60°C. 2-2 Dehydrate and desalt through ethanol series (50% for 5 min, 80% for 1 min, and 96% for 1 min), and air dry. *on membrane filter* 2-1 Filter the samples through membrane filters. 2-2 Dip the filters in 0.1–0.2% low-melting-point agarose, place them upside-down on Parafilm, and dry at 35–60°C. 2-3 Place ethanol on the filters to detach from Parafilm, and air dry. 2-4 Cut the filters, if necessary ***Permeabilization*** 3-1 Lysozyme, lysozyme + achromopeptidase, proteinase K, pseudomurein endopeptidase, HCl, or microwave treatments, based upon your interests. 3-2 Wash in TNT (100 mM Tris-HCl [pH 7.5], 150 mM NaCl, 0.05% Tween 20) or PBSX (0.05% Triton X-100 in PBS) for 5–15 min at room temperature (RT). 3-3 Wash in ultra-pure water for 1 min, dehydrate in ethanol for 1 min, and air dry. ***Endogenous peroxidase inactivation*** 4-1 0.15% H_2_O_2_ in methanol, diethylpyrocarbonate, and/or HCl treatments depending on your samples. 4-2 Wash in TNT or PBSX for 5–15 min at RT, if necessary. 4-3 Wash in ultra-pure water for 1 min, dehydrate in ethanol for 1 min, and air dry. ***In situ hybridization and washing*** 5-1 Prepare hybridization buffer: *e.g.*, 0.9 M NaCl, 20 mM Tris-HCl [pH 7.2], 10% dextran sulphate, 1% blocking reagent, 0.01% SDS, and formamide (concentration depends on the probe) 5-2 Mix probe with hybridization buffer at a final concentration of 0.1 μM. 5-3 Incubate at 35–46°C for more than 2 hours. 5-4 Prepare washing buffer: *e.g.*, 0.9 M NaCl, 20 mM Tris-HCl [pH 7.2], 5 mM EDTA, 0.01% SDS, and formamide. 5-5 Dip the glass slides or the filters in washing buffer and incubate for 10–20 min at 2°C above the hybridization temperature. ***Catalyzed reporter deposition (tyramide signal amplification)*** 6-1 Dip the glass slides or the filters in TNT or PBSX for 15 min at RT. Do not air dry. 6-2 Prepare tyramide working solution: *e.g.*, one volume of tyramide and 50–100 volumes of amplification buffer (0.0015% H_2_O_2_, 0.1% blocking reagent, 10–20% dextran sulphate in PBS) or if using a kit from PerkinElmer, one volume of tyramide, 37.5 volumes of amplification diluent, 12.5 volumes of 40% dextran sulphate, and 0.5 volumes of 10% blocking reagent. 2 M NaCl and *p*-Iodophenylboronic acid (20:1 to tyramide concentration) can be added to further enhance signals. 6-3 Incubate with tyramide working solution for >15 min at 37°C. 6-4 Wash in TNT or PBSX for 15 min at RT or elevated temperature of 45–55°C. 6-5 Wash in ultra-pure water for 1 min, dehydrate in ethanol for 1 min, and air dry.

**Table 3:** Comparison of oligonucleotides and polynucleotides

	Oligonucleotides	Polynucleotides
Specificity	**Higher**. Single mismatch can be distinguished with or without competitor probes (depends on sequences and hybridization conditions).	**Lower**. The threshold for discrimination using polynucleotide probes has been reported to have 70–89% sequence identity.
Sensitivity	**Lower.** The number of molecules to be labeled is low (usually one or two).	**Higher.** Probes can be labeled with many molecules.
Design flexibility	**Higher**. Probes can be designed for a conserved region of a gene and also for a species-specific region.	**Lower**. Probe sequences depend on the template DNA.
Commercial availability	**Yes**. Fluorescently-, hapten-, and HRP-labeled probes can be purchased.	**No.** Probes need to be generated via *in vitro* transcription or PCR.
Permeability (nucleotides only)	**Higher**.	**Lower.**
Definition of Tm (Dissociation behavior)	**Sequence-dependent.** The point at which half the oligonucleotides are dissociated.	**Base content-dependent**. The point at which half the base pairs are dissociated.
